# Experimental Investigation and Damage Identification of High-Pile Wharf Framed Bents under Horizontal Impact Loads

**DOI:** 10.3390/s24020563

**Published:** 2024-01-16

**Authors:** Yonglai Zheng, Fei Xiao, Ruxue Zhang, Tanbo Pan, Xin Lan, Xubing Xu, Chenyu Hou

**Affiliations:** College of Civil Engineering, Tongji University, Shanghai 200092, China; zyll@tongji.edu.cn (Y.Z.); shxf1977@163.com (F.X.); 1830149@tongji.edu.cn (T.P.); 2232620@tongji.edu.cn (X.L.);

**Keywords:** high-pile wharf, ship impact load, damage properties, dynamic response, damage identification

## Abstract

This study investigates damage characteristics, dynamic structural performance changes, and quantitative damage assessment of high-pile wharf framed bents exposed to horizontal impact loads. Through extensive testing of wharf framed bents under such loads, a damage identification approach based on stiffness, natural vibration period, and acceleration data derived from experiments is presented. The findings reveal that under horizontal impact loads, framed bents initially exhibit tensile damage and leaning piles, followed by short straight piles. Additionally, structural damage results in a reduced self-oscillation frequency and an increased amplitude decay rate. Both stiffness-based and cycle-based damage indicators effectively track the cumulative damage progression of the structure. However, the cycle-based damage indicators demonstrate superior stability and accuracy, while acceleration-based indicators precisely identify the moment of damage mutation. This research contributes to enhancing local components, implementing damage identification methods, and advancing health monitoring practices in high-pile wharf projects, aligning with the standards of scientific publications in the field.

## 1. Introduction

High-pile wharfs are widely used in various ports due to their high load-bearing capacity and strong applicability. Due to the complex natural environment and working conditions, many high-pile wharfs currently being used have experienced partial and overall damage of varying degrees during long-term service. It is of great significance to understand the damage properties of terminals and determine the extent of damage for clarifying the state of terminal health and ensuring port safety. Based on the existing research, the damage to high-pile wharfs mainly comes from horizontal loads, vertical loads, and environmental loads. Among them, the horizontal impact load caused by ship berthing is an important parameter in the structural design of high-pile wharfs, which is also the main factor of affecting safety of wharf [1,2,3,4,5].

Scholars have conducted extensive research on damage to high-pile wharf foundation piles caused by horizontal impact loads. In actual high-pile wharf projects, the pile foundation and beams are mostly cast in concrete. This method, which is similar to a rigid connection, makes it possible for the top deflection of the straight pile to be maximized under a static horizontal load, making it easy to cause damage [6]. The results of the horizontal impact test of single straight piles show that [7] damage to straight piles mainly occurs in the form of plastic hinges, and the longer and larger piles are more likely to be damaged. This is because the impact energy is mainly converted into the deformation energy of the foundation pile during the entire impact process and concentrated in the plastic hinge area [8]. For inclined piles, under the action of horizontal impact load, the maximum negative bending moment occurs at the top of the inclined pile, and the maximum positive bending moment occurs at the interface between the pile and the soil [9]. There are differences in the static and dynamic properties of piles with different inclination angles. By optimizing the inclination angle of inclined piles, it is possible to effectively change the stress distribution and damage degree of inclined piles under the same load [10].

In the realm of damage identification of foundation piles in the context of high-piled wharf structures, several studies have made significant contributions to understanding the dynamic properties and response characteristics when subjected to various impact loads. Liu et al. [11] collected stress response data of pile tops to describe the dynamic properties of high-piled wharfs under ship impact. Similarly, Zhao et al. [12] drew the stress–time curve of the foundation pile, and studied the dynamic response properties of pier under impact load by extracting the curve properties. In terms of local dynamic properties, the modal strain energy method is also recognized as a method that can determine damage [13,14,15]. Hadjian et al. [16] proposed a damage identification method based on modal kinetic energy change rate as a parameter, which can simultaneously locate and quantify damage. In order to accurately determine damage, Shu et al. assessed pile damage by arranging strain gauges in pairs on foundation piles and using changes in strain curvature [17]. Shu et al. [18] used optical fiber grating sensors to accurately measure the deflection of foundation piles before and after damage and proposed a method for identifying foundation pile segmented damage. Based on modal curvature, Wang et al. [19] analyzed the influence of each node on the overall CMD curve shape through a data deletion model and established statistical characteristic indicators reflecting the difference between damaged units and undamaged units. Khie et al. [20] derived and used frequency equations and display expressions for frequency response functions to identify and evaluate damage.

The above-mentioned studies have separately researched the stress properties, damage properties, and displacement of straight piles and inclined piles under horizontal impact loads. They also proposed damage calculation and estimation methods based on pile top displacement and foundation pile strain. However, for the high-pile wharves containing straight piles and inclined piles, there is insufficient research on the sequence of damage to pile foundations without providing any clear damage assessment. At the same time, most of the above damage identification methods require arranging a large number of sensors to collect static and dynamic data of foundation piles. However, it is highly difficult to implement this method under the complex working conditions of high-pile wharf.

The accumulation and development of damage to foundation piles not only reduces their mechanical properties, but even causes complete failure, which ultimately leads to the redistribution of stress on the dock foundation piles and changes in the dynamic performance of the overall structure. In the design specifications for port engineering structures, the horizontal displacement of the wharf is an important factor in determining whether the structure is unstable. Therefore, scholars mostly use the change in horizontal displacement of the wharf as the dynamic response index of the wharf. Liu et al. [21] theoretically derived the displacement force amplification coefficient under the impact of ship impact load and analyzed the dynamic response properties of the wharf through the amplified displacement time history data. Wang et al.’s [22] horizontal load test showed that the dynamic response of a fully straight pile terminal could be obtained by multiplying the static calculation results obtained by the P-Y curve method by the amplification factor. The above studies have proved the effectiveness of displacement as a response parameter for reaction kinetics, but there were large differences in the obtained dynamic amplification coefficients, meaning that no unified opinion could be reached.

In terms of overall damage identification, Xie et al. [23] proposed that the overall damage of the dock could be estimated through changes in parameters based on the method of probability theory. In this method, the damage to the wharf caused by various factors can be comprehensively considered without sufficient precision in damage identification. Sun et al. [24] used modal analysis to study the dynamic response of high-pile wharves under environmental excitation, and to explore the application of environmental excitation loads to the structure to identify structural damage. Zhang et al. [25] proposed a damage diagnosis index using the absolute value of the residual force vector based on the vibration differential equation and verified the effectiveness of the method through numerical simulation. Cao et al. [26] proposed a nondestructive detection method for foundation pile stress reflection waves based on optimal fundamental wavelet continuous wavelet transformation. This method can effectively improve weak damage detection accuracy. Furthermore, Xie et al. [27] conducted comprehensive damage identification research on the structure of Pier 2 in Yangzhou Port’s Jiangdu Port Area. The approach involved on-site inspection, numerical analysis, and vibration testing, providing insights into changes in dynamic characteristics under different damage scenarios. Qian et al. additionally [28], introduced a novel damage identification method based on the Kent chaotic artificial bee colony algorithm. This method utilized modal shapes and structural natural frequencies as indicators for damage identification of high-pile wharf braces under various operating conditions, demonstrating higher effectiveness compared to particle swarm optimization algorithms. Zhang et al. [29] utilized curvature mode, curvature mode difference, and wavelet transform as indicators for damage identification of high-pile wharves, comparing and verifying the identification effectiveness of these three methods under diverse operating conditions. Li et al. [30] proposed a method for obtaining modal parameters of the complete structure under normal circumstances, suggesting a fitting approach for modal curvature under structural damage conditions. Using the fitted modal curvature as an indicator, they applied support vector machines for damage identification of high-pile wharf piles, validating the effectiveness of this method through practical examples.

The aforementioned research indicates a predominant focus on local damage in studies pertaining to high-pile wharves subjected to horizontal impact loading. Specifically, emphasis has been placed on damage to foundation piles, resulting in a somewhat inadequate exploration of overall wharf damage. Concurrently, the application of numerous sensors on wharf components to detect local damage is deemed impractical in engineering scenarios. This paper introduces and compares three methods that employ a limited number of sensors to assess overall wharf damage and safety effectively. Notably, previous studies often employed a single large impact force during damage tests, neglecting the comprehensive depiction of the damage development process in piles. This paper addresses this gap by recording and analyzing the detailed process of pile damage. The foundation piles of the high-pile wharf model were simplified using similarity theory [31] and “m” [32,33], aligning with limited test conditions to simulate real-world scenarios. Considering that the longitudinal beams of piers enhance structural integrity and increase longitudinal stiffness without affecting the individual stress properties of each pile, a single-sided rack was chosen as the focal point for an indoor experiment under horizontal impact loading [34]. The results reveal that the proposed methods effectively capture the cumulative damage process of the rack, quantifying the degree of damage and offering valuable guidance for practical engineering structural damage assessment and monitoring. Nevertheless, each of the three methods exhibits distinct advantages and disadvantages. This paper recommends employing all three damage indicators simultaneously to accurately assess the degree of damage. Furthermore, to enhance the precision of damage indicators, it is essential to conduct in-depth confirmatory research through preset damage tests on these indicators.

## 2. Experimental Setups

### 2.1. Engineering Background

**Figure 1:**
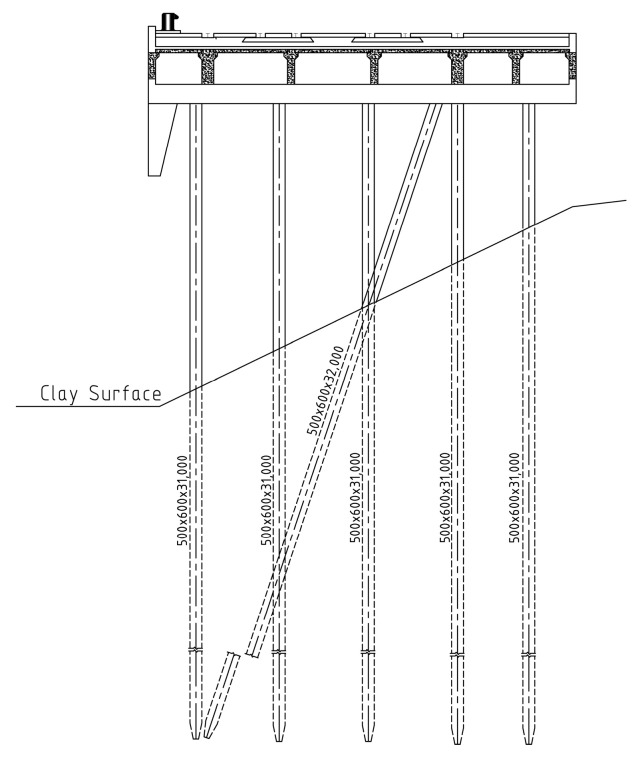
The prototype of a typical single-row rack.

The engineering context for this experiment is a wharf situated on Junggong Road in Shanghai. The prototype of a typical single-row rack for this dock is shown in the Figure 1.

The structural design adopts a permeable framework, with individual rows measuring 33.5 mm in height, 18.0 m in length, and 0.9 m in width. The concrete utilized is of grade C30. For the foundation piles, 600 mm × 600 mm prestressed reinforced concrete square piles are utilized, with a total of 6 piles arranged beneath the framework. These piles are sequentially numbered from 1# to 6# from the front end of the beam to the end of the beam. The pile lengths are computed using the “m” method as prescribed in the port engineering load specifications, and the results of these calculations are displayed in Table 1. The beam itself has a width of 0.9 m, with a prefabricated height of 2.400 m. The ship-side structural element stands at a height of 3.1 m, with dimensions of 0.9 m in width and 1.2 m in length. The designated ship type for this design is a 20,000-ton bulk carrier. The parameters and thicknesses of the soil layers at the prototype terminal are detailed in Table 2.

### 2.2. Model Design

The theory of similarity combines the advantages of mathematical analysis and experimental methods, offering a scientific approach to studying and addressing engineering problems. Model testing plays a crucial role in similarity methods, and when conducting scaled-down model experiments, numerous challenges must be addressed. These challenges include the design and fabrication of scaled models, as well as the derivation of prototype results from model outcomes.

The theory of similarity mainly consists of three parts: the positive theorem of similarity, the PI theorem of similarity, and the inverse theorem of similarity. Based on the above three theorems, this experiment uses dimension analysis to derive the similarity criterion and finally calculates the scale of the model. The parameters involved in this study are density, geometry, impact force, stress, cross-sectional area, stiffness, acceleration, and time. In this research, important fundamental parameters, such as length (L), elastic modulus (E), and density (ρ), have been selected as key variables. All other parameters are quantified in terms of mass (M), geometry (L), and time (T), as shown in Table 3.

In this research, important fundamental parameters, such as length (L), elastic modulus (E), and density (ρ), have been selected as key variables. The dynamic system equations for similar models have been established, and the principles of Buckingham’s π theorem, the second theorem of similarity, have been applied to determine the dimensionless similarity numbers that relate different dimensional quantities. This methodology aids in gaining a better understanding of engineering problems and provides an effective means of investigating and solving them. 

The similarity ratios are given by $\lambda_{L}=1/10$, $\lambda_{E}=1/5$ etc, $\lambda_{\rho }=1/1$, respectively. 

Subsequently, the remaining variables can be expressed as functions of L, E, and ρ. The similarity criteria can be obtained from dimensional analysis. The models are similar, as shown in Table 4 [35]

Based on the derived similarity ratios, this study employed particle concrete and galvanized iron wire for constructing the model framework. Particle concrete and galvanized iron wire have found extensive use in similar model experiments [36]. Numerous numerical simulation studies have demonstrated their effectiveness in simulating the properties of concrete and reinforcement steel. Drawing from referenced experimental research [37], this experiment selected the particle concrete labeled as C8. Table 5 presents the material composition ratios for this C8 particle concrete.

It is worth noting that the diameter of the galvanized iron wire is determined based on the principle of equivalent strength, as per the following Equation (1):

(1) $$\frac{A_{ps}f_{ps}}{A_{pc}f_{pc}}=\frac{A_{ms}f_{ms}}{A_{mc}f_{mc}}$$

where $A_{ps}$ and $f_{py}$ are expressed as the effective longitudinal section area and steel bar strength in the prototype structure, respectively; $A_{pc}$ and $f_{pc}$ are the effective longitudinal section area of concrete in the prototype structure; $A_{ms}f_{my}$ and $A_{mc}f_{mc}$ are the parameters of granulated concrete and galvanized steel wire in the corresponding model, respectively. Through laboratory configuration and measurement, the parameters of micro-particle concrete are $E_{mc}=1.5\times 10^{4}\;N/mm^{2}$ and $f_{mc}=8.7\;N/mm^{2}$. The parameters of galvanized steel wire are $E_{ms}=2.06\times 10^{5}\;N/mm^{2}$ and $f_{ms}=4.2\times 10^{2}\;N/mm^{2}$.

For the pile length simplified by the “m” method, the length of the pile in the model test is obtained based on the above similarity criterion. The similar results are shown in Table 6.

Considering the different lengths of foundation piles and the slope conditions of pier, the model base is made using the same model concrete.

### 2.3. Test System

**Figure 2:**
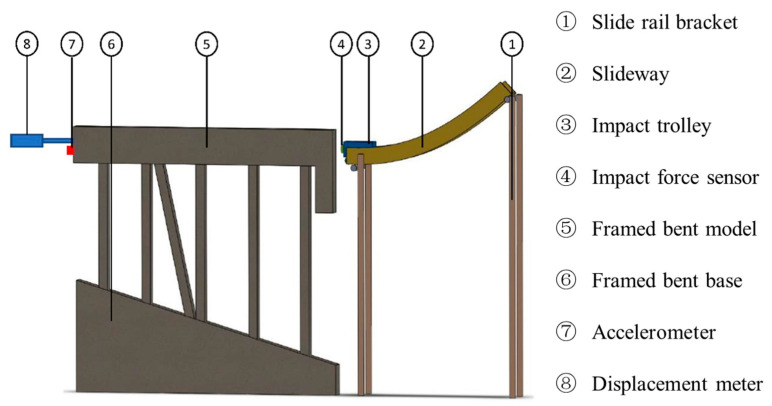
Model test system.

**Figure 3:**
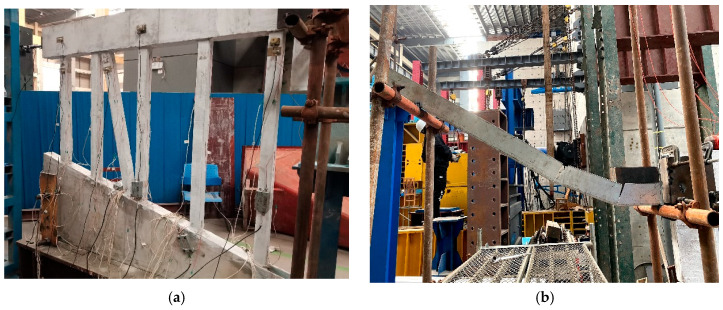
Test equipment. (**a**) Impact device; (**b**) frame model.

Figure 2 illustrates the test system for simulating ship impact on a high-pile wharf framework, comprising an impact trolley, rails, the framework model, and testing equipment. In Figure 3a, the impact device consists of rails and an impact trolley. The impact trolley has a mass of 10 kg and dimensions of 0.2 m in length, 0.1 m in width, and 0.15 m in height. The rails have an inner width of 0.12 m, an outer width of 0.14 m, and side plates with a height of 0.10 m, constructed from galvanized steel plates with a thickness of 0.01 m. Figure 3b showcases the framework model after pouring. To restrict movement in all directions, all six degrees of freedom of the framework base are entirely constrained.

**Figure 4:**
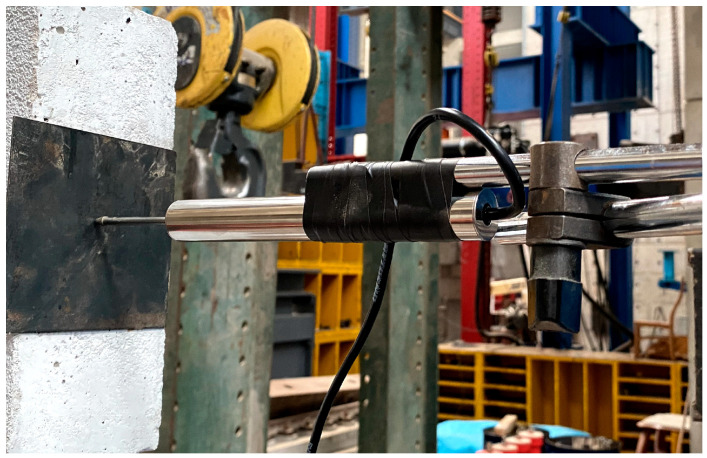
LVDT displacement sensors.

An LVDT displacement sensor was installed at the end of the bending frame in this test, as shown in Figure 4. The sensor has a range of 0–100 mm. The height of the displacement sensor is the same as that of the impact point, allowing it to monitor the horizontal displacement variation in the entire bending frame. A uniaxial accelerometer sensor is mounted directly below the displacement sensor, with a sampling frequency of 1000 Hz.

### 2.4. Experimental Process

This test does not study the damage at the impact location of the rack. The impact area of the rack is reinforced with 10 mm thick rubber pads and steel plates, and the sides of the impact area are also reinforced with steel plates to prevent the spalling of particulate concrete. A scaffolding is set up in front of the rack. The scaffolding is made of 4 vertical poles and 2 horizontal poles. The bottom of the slide rail and the horizontal poles are fixedly connected by electric welding. According to the research purpose, we set up 4 groups of different working conditions, as shown in Table 7.

In order to reduce the test error, the car slid along the rail at the same height and hit the rack 99 times. Every 3 impacts were divided into an impact group, and this set of data was averaged. A total of 33 impact group data were obtained. We named the different impact groups in the manner L-10-1. L indicates impact, 10 indicates that the car’s slip height is 10 cm, and 1 indicates the first impact group under this working condition.

## 3. The Method of Pile Damage Identification Based on Static Strain

The equation for damped free vibration with a single degree of freedom is as follows:

(2) $$\;m\ddot{y}+c\dot{y}+ky=0$$

where *m* represents mass, *c* represents damping, *k* represents structural stiffness, and *y* represents vibration displacement.

According to material mechanics, the bending stiffness of pile is the product of the elastic modulus of material and inertial cross-sectional area. The damage to the foundation piles is mainly shown as a reduction in local effective cross-sectional area due to the development of cracks and a decrease in elastic modulus due to the carbonization of concrete or corrosion of steel bars. Therefore, this study represents foundation pile damage in the form of stiffness reduction. To quantify damage, we define the degree of structural damage based on stiffness $D_{k}$, as follows:

(3) $$D_{k}=1-\frac{k_{D}}{k_{0}}$$

where $k_{D}$ is the stiffness after the foundation pile is damaged, and $k_{0}$ is the stiffness when the foundation pile is not damaged. Based on Formula (1), the vibration period of structure T can be obtained from the following Equation (4):

(4) $$T=\frac{2\pi }{\omega }$$

The circular frequency before and after damage ω is obtained by using the following Equation (5):

(5) $$\omega =\sqrt{\frac{k}{m}}$$

Damping ratio ξ:

(6) $$\xi =\frac{c}{2m\omega }$$

According to structural mechanics, the stiffness of a structure can be expressed as the slope of load and displacement. In static tests, it can be calculated by the ratio of measured force and displacement. Stiffness is an inherent property of structures and materials that does not change with the magnitude of the load. In dynamic experiments, when the structure or component is not damaged, its stiffness remains unchanged at any time. Therefore, the displacement and load data when the structural velocity is 0 can be used to calculate the structural stiffness.

**Figure 5:**
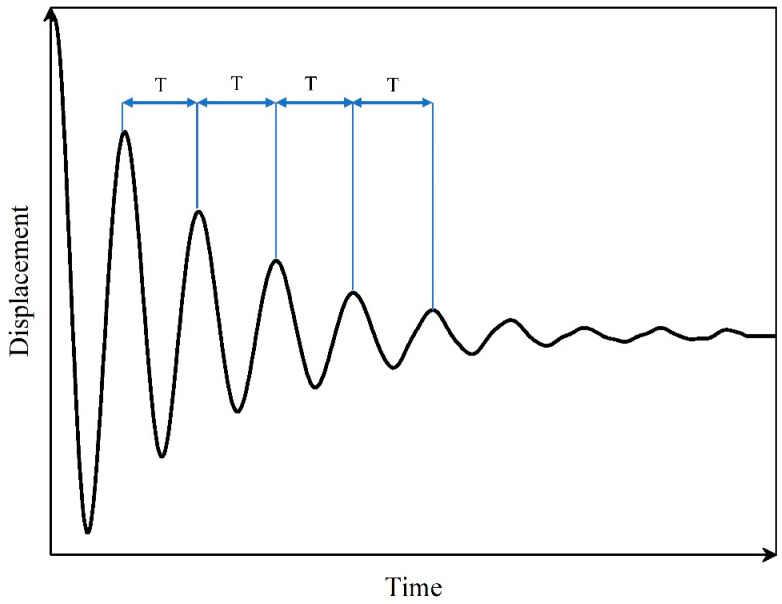
Typical natural vibration curve.

A typical damped free vibration curve Is shown in Figure 5. The interval time between adjacent wave peaks is the period T, the total free vibration time t=nT, and n means that there are n vibration periods. The measured vibration period can be obtained from the ratio of total free vibration time to vibration times. Assuming that the minor damage to structure does not cause a change in its quality, Equations (4) and (5) are brought into Equation (3) to obtain the degree of structural damage based on the cycle $D_{T}$, as follows:

(7) $$D_{T}=1-\frac{T_{0}^{2}}{T_{D}^{2}}$$

where $T_{0}$ represents the self-oscillation period of the structure when $T_{D}$ is not damaged, and the self-oscillation period of the structure after damage.

In the dynamic experiment, the acceleration data can well reflect the vibration properties of the structure. The frequency and amplitude of vibration can be obtained by Fourier transform of acceleration f data A. Defining damage indicators based on acceleration frequency $D_{a}$ can be obtained using frequencies f, as follows:

(8) $$D_{a}=1-\frac{f_{D}^{2}}{{f_{0}}^{2}}$$

## 4. Results and Discussion

### 4.1. Damage of Pile

**Figure 6:**
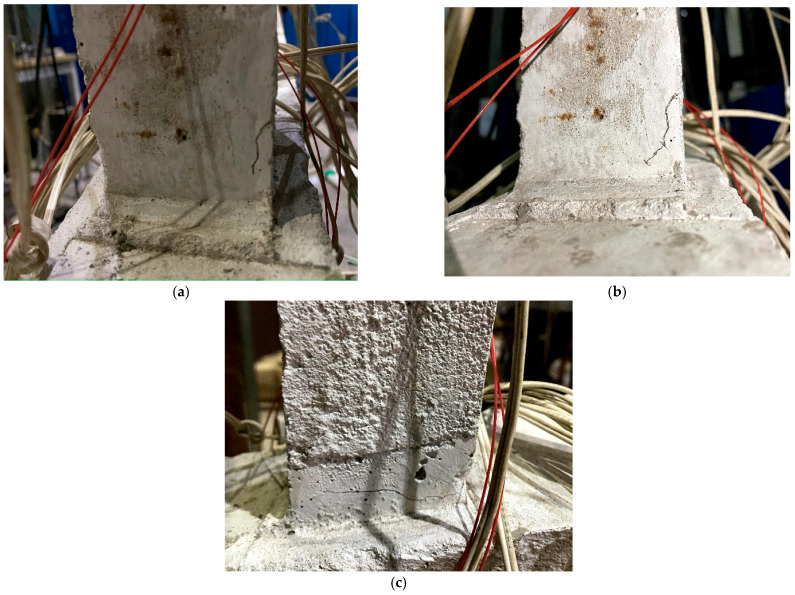
Damage of the 1# pile under condition L-30. (**a**) L-30-11; (**b**) L-30-22; (**c**) L-30-33.

**Figure 7:**
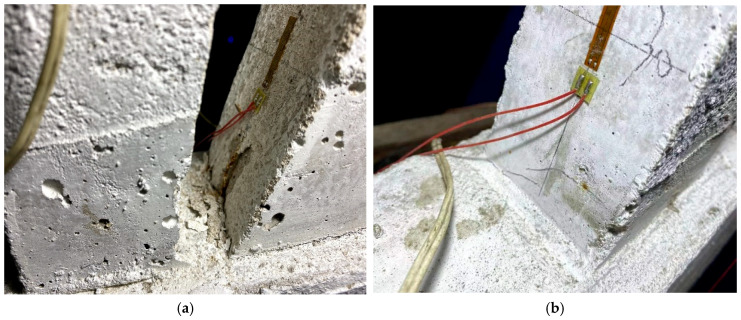
Damage of the 4# pile under condition L-40. (**a**) L-40-11; (**b**) L-40-22.

**Figure 8:**
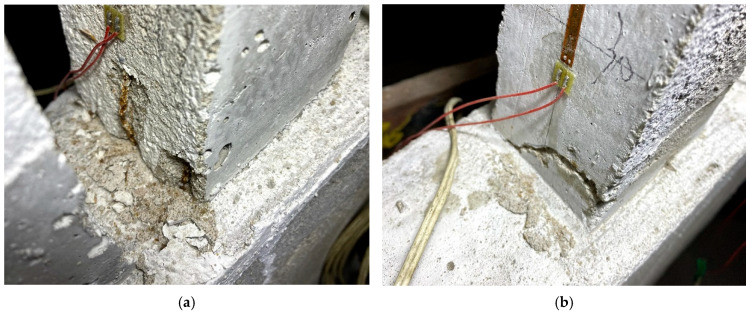
Damage of the 4# pile under condition L-40. (**a**) L-40-22; (**b**) L-40-33.

Following the completion of each impact group, a thorough examination of each pile was conducted to identify any cracks. Subsequent testing revealed that, following the impact of L-30-11, a 15 mm dipping crack appeared at the base of the 1# straight pile, as illustrated in Figure 6a. The impact from L-30-22 resulted in the ongoing development of the crack at the bottom of pile 1#, as depicted in Figure 6b. After the impact of L-30-33, a horizontal crack appeared on the outside of pile 1, as shown in Figure 6c. For the L-40-11 impact group, the rebar at bottom of the 4 # inclined pile bent and exposed a concrete crack at the joint of pile and foundation base, as shown in Figure 7a. For the L-40-22 impact group, the 4 # inclined pile’s damage became intensive, as shown as in Figure 7b. At the same time, the bottom of the 6 # straight pile showed a crack, and after the L-40-33 impact groups, the length and width of this tensile crack both increased, as shown in Figure 8a,b.

It can be seen from the above results that, due to the low tensile strength of micro-concrete, the pile damage mainly occurs in the form of tensile cracks in the concrete. The 1# pile has an oblique crack because the test fails to completely limit the lateral displacement of the upper part of the rack. The car inevitably generates lateral impact force during the impact process, which eventually lead to a lateral pull crack. The failure of the 4# inclined pile indicates that the inclined pile predominantly withstands the horizontal impact force. The pile experiences significant tensile stress at the bottom, surpassing the tensile strength of the particulate concrete. Consequently, this leads to tensile damage in the concrete, causing the wire to stretch and bend, ultimately becoming exposed. At the same time, the pile failure sequence changes from inclined piles to short straight piles. After the inclined pile is damaged, the internal force of the rack is redistributed, the ability of the inclined pile to resist horizontal displacement is reduced, and the ability of the straight pile to resist horizontal displacement is increased. Under the same horizontal load, piles with lower flexibility are damaged first.

### 4.2. Displacement-Based Structural Dynamic Properties

**Figure 9:**
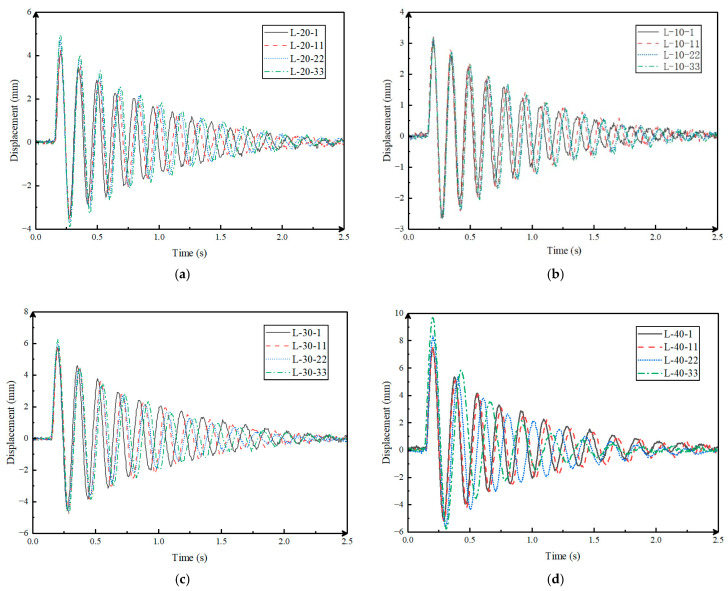
Displacement of natural vibration. (**a**) L-10 representative group; (**b**) L-20 representative group; (**c**) L-30 representative group; (**d**) L-40 representative group.

Displacement is the most direct response of a structure after bearing load. Structural damage has an important impact on the dynamic displacement time course curve and is limited by space. This chapter uses impact groups 1, 11, 22, and 33 under various working conditions as examples to draw the displacement time course curve. The results of the drawing are shown in Figure 9.

Under the L-10 working conditions, the total vibration of each impact group is not changed, which is 2.25 s. However, as the number of impacts increased, there was a clear difference in the timing of the peak occurrence of each curve. The vibration curve properties of L-20 and L-30 working conditions are the same as those of L-10 working conditions. Under L-40 working conditions, from the low impact group to the high impact group, the structural vibration is decreased over time, the rate of amplitude reduction is increased markedly, and the difference in time for each curve peak to occur is even more significant. At the same time, the number of peaks in the vibration process decreased from 13 in L-40-1 to 9 in L-40-33.

According to the cycle calculation method in Chapter 3, the average self-oscillation period of the impact group under various working conditions and the period difference with the No. 1 impact group are calculated. The calculation results are listed in Table 8.

As seen in Table 8, the self-oscillation period of the rack gradually increases along with the number of impacts is increased. It can be assumed that damage to the frame caused an increase in the self-oscillation cycle of the structure. Under low loads, such as L-10 and L-20, the self-oscillation cycle is increased by 1.3% and 1.9%, respectively. Under high loads, such as L-30 and L-40, the self-oscillation periods are increased by 9.8% and 33%, respectively.

Corresponding to the rack damage process in Section 3, the above results show that the effect of rack damage on the free vibration curve is mainly reflected in the vibration cycle and duration. Damage to the structure can be equivalent to a reduction in stiffness. As seen from Equations (3) and (4), a decrease in stiffness causes a decrease in the frequency of the structural mode, thereby causing an increase in the self-oscillation period. The result is that the number of structural vibrations per unit time is reduced. At the same time, the vibration duration of the structure is affected by its damping ratio. It can be seen from Equations (3) and (5) that the damping ratio of the structure is positively correlated with the period. An increase in the damping ratio of the structure means an increase in the energy dissipation rate, which is manifested in an increase in the amplitude reduction rate of the structure in this chapter and a decrease in the vibration duration. This fully shows that the displacement of the structure can reflect the damage state of the structure to a certain extent, but it cannot quantify the degree of damage.

### 4.3. Acceleration-Based Structural Dynamic Properties

**Figure 10:**
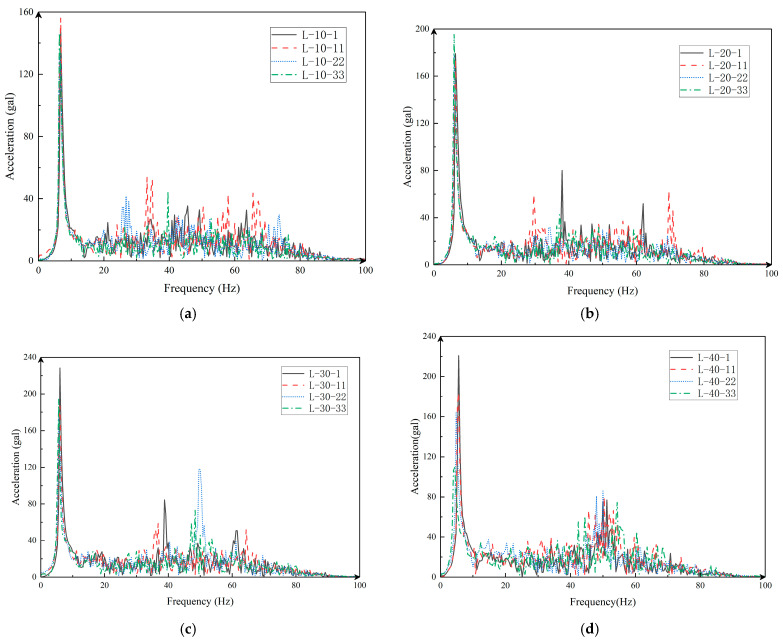
Fourier spectrum. (**a**) L-10 representative group; (**b**) L-20 representative group; (**c**) L-30 representative group; (**d**) L-40 representative group.

Acceleration is one of the most important parameters in structural dynamics. Through Fourier transform of acceleration data, the dynamic properties of structure before and after damage can be obtained. Research by many scholars shows that the lower-order modality of the structure can already fully reflect its kinetic properties. This chapter selects first-order modal data as the subject of study. Also select the impact groups in Section 3 and draw Fourier spectra for the different impact groups under each working condition, see Figure 10.

From Figure 10a–c, it can be seen that under the L-10, L-20, and L-30 conditions, the first-order vibration frequencies of each impact group are almost the same, and the high-order frequency distribution is relatively messy. Figure 10d shows that as the number of impact groups increases, the first-order frequency decreases, and the higher-order frequency distribution tends to be concentrated. Upon comparing the first-order amplitudes of each working condition, it can be found that as the impact load increases, the first-order amplitude increases, but the amplitude growth rate decreases. Combined with the rack damage process in Section 3, the first-order vibration frequency of the structure decreases as the degree of damage to the rack increases. At the same time, although the higher-order frequency distribution of the structure becomes more concentrated as the degree of damage increased, there is no obvious distribution rule.

In Table 9, the frequency of impact groups under various working conditions is listed. The frequency precision after the Fourier transform is determined by the sampling rate and sampling time, which is 0.4 Hz in this experiment.

The frequency difference percentage in Table 8 shows that under various working conditions, the first-order frequency of the row frame is decreased as the number of impact groups is increased, indicating that the row is damaged. An impact group with reduced frequency can be used to determine the moment when the cumulative damage changes abruptly. Under the L-40 working condition, the frequency is decreased by 5.8% and 11.6%, respectively, indicating that the structure had an obvious damage mutation between impact groups 11, 22, 22 and 33, respectively.

### 4.4. Damage Index

**Figure 11:**
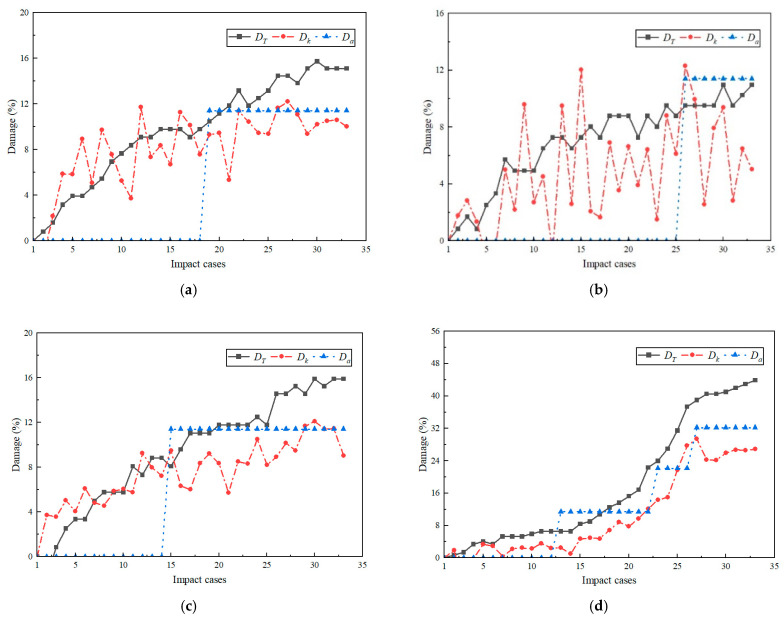
Damage index. (**a**) L-10; (**b**) L-20; (**c**) L-30; (**d**) L-40.

Damage indicators based on measured stiffness, damage indicators based on displacement cycles, and damage indicators based on acceleration frequency are expressed by $D_{k}$, $D_{T}$, and $D_{a}$ separately. The calculation results of the damage indicators of all impact groups under each working condition are plotted in Figure 11.

The stiffness in this test is determined by the peak impact force and peak displacement. Due to the short duration of the impact, there is an error in the peak impact force obtained by the test due to the sampling precision of the impact force sensor. At the same time, the displacement of the structure is at the millimeter level, which leads to a large error in the calculated stiffness. The response in the curve of Figure 11 shows that, for $D_{k}$, the discrete type of value is larger. However, under a higher impact load (the L-40 working condition) $D_{k}$, the error decreases, enhancing the quasi-determination of the degree of damage. The properties of Formula (6) show that for $D_{T}$, the growth rate decreases with the increase in T0, indicating that a smaller period difference can obtain a larger damage index difference. The cycle of this test comes from the average value of the cycle during the full self-oscillation period of the rack. Taking the L-10 working conditions in Table 7 as an example, the L-10-22 cycle is increased by 0.6% compared to the L-10-1 cycle, and the degree of damage is increased by 3.8%. This led to a rapid $D_{T}$ increase in the number of impacts as the number of impacts in Figure 11 increased. By L-40-33, the damage index reached 46%, which is much larger than the damage index based on stiffness calculation. At the same time, due to the high sensitivity of this damage index, the process of cumulative damage in the rack can be well described.

The Fourier transform of acceleration can obtain the frequencies of each order of free vibration of structure. The identification precision of frequency in this test is 0.4 Hz, and the $D_{a}$ curve in Figure 10 exhibits obvious segmented properties with a difference of 5.8%. Figure 10 shows that the increase in impact force causes damage to occur earlier, as shown $D_{a}$ by mutations L-10-25 to L-20-17 and L-30-15. At the same time, L-40 showed obvious mutations in working conditions of 13, 22, and 27, respectively. This indicates that in the L-40-13, L-40-22, and L-40-27 impact groups, there was serious damage to the shelves. When comparing $D_{T}$ to $D_{a}$, $D_{a}$ is significantly less than $D_{T}$ under the same working conditions. This discrepancy arises because, for $D_{a}$, the first-order frequency was employed, neglecting the contribution of other modes, and for $D_{T}$, the self-oscillation period under the influence of the complete mode shape was selected.

The above analysis can indicate that precision of a damage index based on stiffness. $D_{k}$ is affected by the measuring equipment, but it can respond to the damage process of the rack under high damage. The damage index of the displacement-based self-oscillation period $D_{T}$ can respond well to the complete damage process, but the calculation results are too large. The acceleration-based damage index $D_{a}$ cannot accurately reflect the entire process of rack damage, but it can accurately identify the moment when major damage occurs.

## 5. Conclusions

Through the model tests of high-pile wharf framed bents affected by impact loads, the effects of damage on the dynamic properties of the structure are studied with the structural damage indicators based on stiffness, cycle, and acceleration are also discussed. Upon comparing the dynamic parameters of framed bents under different impact loads and calculating different damage indices, the following conclusions can be obtained:

(1) Damage to framed bents under horizontal impact loads mainly occurs at the bottom of the pile, and also occurs in the form of tensile damage. The inclined pile was damaged first. After the damage, the internal forces of the row frame were redistributed, and the damage to the straight piles showed properties from short piles to long piles. At the same time, the lateral force of the impact load will cause long straight piles to first experience lateral tensile damage. In subsequent similar tests, attention should be paid to the lateral restraint of the upper part of the rack.
(2) Damage has a significant effect on the dynamic properties of the rack. The main symptoms are that an increase in damage decreases the self-oscillation period, increases the amplitude attenuation rate, and causes a decrease in first-order frequency after Fourier transformation based on acceleration data. At the same time, the precision of the Fourier spectrum frequency has a significant impact on identifying frequency changes. It is recommended that the sampling time can be increased in the future to increase the frequency precision after conversion.
(3) Damage indexes based on stiffness, self-oscillation period, and acceleration can all be used to quantify rack damage. Affected by the precision of testing instruments, the stiffness data can reflect the damage trend, but they cannot accurately quantify the rack damage. The self-oscillation period of the structure can be easily obtained through a displacement meter, and at the same time, the damage index $D_{T}$ can better characterize the damage properties of the rack and quantify the damage degree, but the calculation results are too large. The damage index $D_{a}$ can effectively identify when cumulative damage has abruptly changed, while damage calculation results based on the first-order frequency are conservative.

## Figures and Tables

**Table 1:** The calculated length of the piles (unit: m).

Pile Number	1	2	3	4	5	6
Calculate Length	14.40	13.00	11.40	12.40	9.80	8.55

**Table 2:** Soil layer parameters of the prototype wharf.

Soil Layer Serial No.	Soil Layer Name	Severe (KN/m^3^)	Adhesion (kPa)	Internal Friction Angle (°)	Soil Thickness (m)
1	Sandy silt	19	5	37	5.8
2	Clay	17.5	11	14.5	7.0
3	Silty clay	19.8	0	19.0	5.9
4	Sandy silt	18.7	5	34.0	9.5

**Table 3:** Dimensional analysis.

Parameter	Symbol	Dimension	M	L	T
Stress	σ	$M^{1}L^{-1}T^{-2}$	1	−1	−2
Force	F	$M^{1}L^{1}T^{-2}$	1	1	−2
Cross-sectional area	A	$M^{0}L^{2}T^{0}$	0	2	0
Density	ρ	$M^{1}L^{-3}T^{0}$	1	−3	0
Stiffness	K	$M^{1}L^{2}T^{-2}$	1	2	−2
Acceleration	a	$M^{0}L^{1}T^{-2}$	0	1	−2

**Table 4:** Model similarity ratio.

Physical Quantity	Similarity Criteria	Similarity Relation	Similarity Ratio
External Force *F*	${\left[L\right]}^{2}{\left[\rho \right]}^{0}{\left[E\right]}^{1}$	$\lambda_{F}=\lambda_{E}\lambda_{L}^{2}$	1/500
Displacements δ	${\left[L\right]}^{0}{\left[\rho \right]}^{0}{\left[E\right]}^{1}$	$\lambda_{\delta }=\lambda_{E}$	1/5
Cross-sectional area *A*	${\left[L\right]}^{2}{\left[\rho \right]}^{0}{\left[E\right]}^{0}$	$\lambda_{A}=\lambda_{L}^{2}$	1/100
Stiffness *k*	${\left[L\right]}^{3}{\left[\rho \right]}^{0}{\left[E\right]}^{1}$	$\lambda_{k}=\lambda_{E}\lambda_{L}$	1/50
Acceleration *a*	${\left[L\right]}^{-1}{\left[\rho \right]}^{-1}{\left[E\right]}^{1}$	$\lambda_{a}=\lambda_{E}/\lambda_{L}\lambda_{\rho }$	2/1

**Table 5:** Mix proportion of micro-concrete.

Rating	Silicate Cement	Sand	Lime	Water
C8	1	6.0	0.6	0.5–0.6

**Table 6:** Calculated length of model piles (unit: m).

Pile Number	1	2	3	4	5	6
Calculate Length	1.44	1.30	1.14	1.24	0.98	0.86

**Table 7:** Test conditions (unit: cm).

Working Conditions	1	2	3	4
Descending Height	10	20	30	40

**Table 8:** Period of natural vibration.

Working Conditions	Impact Group	Period *T* (s)	Period Difference $\Delta_{T}$(s)	Difference Percentage (%)
L-10	1	0.152	0	0
	11	0.152	0	0
	22	0.153	0.001	0.6
	33	0.155	0.002	1.3
L-20	1	0.160	0	0
	11	0.160	0	0
	22	0.162	0.002	1.3
	33	0.165	0.003	1.9
L-30	1	0.166	0	0
	11	0.172	0.006	3.6
	22	0.175	0.009	5.4
	33	0.183	0.017	9.8
L-40	1	0.182	0	0
	11	0.187	0.005	2.7
	22	0.204	0.017	9.3
	33	0.242	0.06	32.9

**Table 9:** First-order frequency.

Working Conditions	Impact Group	Frequency *f* (Hz)	Frequency Difference $\Delta_{f}$ (Hz)	Difference Percentage (%)
L-10	1	6.8	0	0
	11	6.8	0	0
	22	6.8	0	0
	33	6.4	−0.4	−5.8
L-20	1	6.8	0	0
	11	6.8	0	0
	22	6.4	−0.4	−5.8
	33	6.4	−0.4	−5.8
L-30	1	6.8	0	0
	11	6.8	0	0
	22	6.4	−0.4	−5.8
	33	6.4	−0.4	−5.8
L-40	1	6.8	0	0
	11	6.8	0	0
	22	6.4	−0.8	−11.6
	33	6.0	−1.2	−32.2

## Data Availability

The data presented in this study are available on request from the corresponding author.
